# *PIK3R1* negatively regulates the epithelial-mesenchymal transition and stem-like phenotype of renal cancer cells through the AKT/GSK3β/CTNNB1 signaling pathway

**DOI:** 10.1038/srep08997

**Published:** 2015-03-11

**Authors:** Youcheng Lin, Zhao Yang, Abai Xu, Pei Dong, Yi Huang, Huan Liu, Feida Li, Haifeng Wang, Qian Xu, Yongqiang Wang, Da Sun, Yong Zou, Xiaowen Zou, Yu Wang, Duo Zhang, Hongjie Liu, Xun Wu, Meng Zhang, Yu Fu, Zhiming Cai, Chunxiao Liu, Song Wu

**Affiliations:** 1 Department of Urology, Zhujiang Hospital of Southern Medical University, Guangzhou 510280, China; 2 National-Regional Key Technology Engineering Laboratory for Clinical Application of Cancer Genomics, Second People's Hospital, the First Affiliated Hospital of Shenzhen University, Shenzhen 518035, China; 3 CAS Key Laboratory of Infection and Immunity, Institute of Biophysics, Chinese Academy of Sciences, Beijing 100101, China; 4 University of Chinese Academy of Sciences, Beijing 100049, China; 5 BGI-Shenzhen, Shenzhen 518083, China; 6 Department of Pathology, Zhujiang Hospital of Southern Medical University, Guangzhou 510280, China; 7 Department of Urology, Sun Yat-Sen University Cancer Center, Guangzhou 510060, China; 8 The Genome Institute, Washington University in St. Louis, 4444 Forest Park Ave, St. Louis, Missouri 63108, USA; 9 Department of Urology, the Second Affiliated Hospital of Kunming Medical College, Kunming 650101, China; 10 Department of Oncology, the First Affiliated Hospital of Soochow University, Suzhou 215006, China

## Abstract

The phosphoinositide 3-kinase (PI3K)/protein kinase B (AKT) pathway has been identified as an important pathway in renal cell carcinoma (RCC). We have reported a nonsense mutation in *PIK3R1*, which encodes the regulatory subunit of PI3K, in a metastatic RCC (mRCC), while the mutation was absent in the corresponding primary RCC (pRCC). To identify the function of *PIK3R1* in RCC, we examined its expression in normal kidney, pRCC and mRCC by immunohistochemistry and real-time polymerase chain reaction. The expression of PIK3R1 significantly decreased in pRCC and was further reduced in mRCC compared with normal tissue. Besides, its expression levels were negatively correlated with T-category of tumor stage. Additionally, 786-O and A-704 cells with *PIK3R1* depletion introduced by CRISPR/Cas9 system displayed enhanced proliferation, migration and epithelial-mesenchymal transition (EMT), and acquired a stem-like phenotype. Moreover, the *PIK3R1* depletion promoted the phosphorylation of AKT in the cells. The knockdown of *AKT* by shRNA reduced p-GSK3β and CTNNB1 expression in the cells, while the depletion of *CTNNB1* impaired stem-like phenotype of the cells. Overall, *PIK3R1* down-regulation in RCC promotes propagation, migration, EMT and stem-like phenotype in renal cancer cells through the AKT/GSK3β/CTNNB1 pathway, and may contribute to progression and metastasis of RCC.

Renal cell carcinoma (RCC) is one of the most lethal types of urological cancer^1^. Recent studies have markedly increased the understanding of the cell molecular biology of RCC, dominated by the inactivation of *VHL* in ubiquitin-mediated proteolysis pathway (UMPP) and alteration of *PBRM1* involved in chromatin regulation^2^,^3^,^4^. The increased understanding of RCC biological pathways has led to the development of molecularly targeted therapeutic agents that have improved patient outcomes1. However, the advanced and metastatic RCC (mRCC) remains incurable^5^, therefore further studies are highly needed to understand the mechanisms of the molecular basis of resistance and response, thus leading to the discovery of novel targets for the treatment of mRCC.

In addition to UMPP^3^, the phosphoinositide 3-kinase (PI3K)/protein kinase B (AKT) pathway has also been identified as an important pathway in RCC^2^,^6^. The PI3K/AKT pathway begins with the involvement of growth factors binding to the receptor tyrosine kinases^7^. PI3K is activated through attachment to receptors anchored on plasma membrane and generates phosphatidylinositol-3-phosphate (PIP3) by phosphorylating phosphatidylinositol 4, 5-bisphosphate^8^. Through a pleckstrin homology domain, AKT binds to PIP3 and is phosphorylated to pAKT^8^. Class IA PI3Ks are heterodimers that consist of a catalytic subunit (p110α, p110β and p110δ) and a regulatory subunit (p85α, p55α, p50α, p85β, and p55γ)^9^. The catalytic subunit p110α is encoded by *PIK3CA*, while the regulatory subunit p85α is encoded by *PIK3R1*^9^. The PI3K/AKT pathway regulates many aspects of cellular functions, including cell growth, proliferation, translation and survival, and is also involved in pathological conditions^6^. Deregulation of the pathway can lead to disturbance of cell growth, proliferation and survival, thus resulting in growth advantage and metastatic competence of cancer cells^10^.

The alteration of PI3K/AKT pathway has been found in a broad spectrum of cancers^9^. Members of the pathway such as *PIK3CA* and *PTEN* are frequently altered in RCC^2^. Since the pathway plays an important role in RCC pathogenesis^2^, it has been showing a great promise for molecularly targeted treatment of RCC^6^,^9^. However, only a small number of patients benefit from single-agent PI3K targeted therapy11. The related mechanism of unsatisfied effect of PI3K targeted therapy remains to be clarified^11^. Can, in addition to *PIK3CA* and *PTEN*, other member of the pathway play a role and be a target of therapy in RCC?

Accumulating studies have showed an important role of *PIK3R1* in human carcinogenesis^8^,^12^,^13^,^14^. *PIK3R1* has been reported as an oncogene in ovarian and colon tumors^15^, whereas it has been shown as a tumor suppressor in hepatocellular carcinomas^16^. The underexpression of PIK3R1 has been reported to be associated with poor prognosis of breast cancers^17^. A missense mutation of *PIK3R1* which resulted in decrease of PIK3R1 expression has also been strongly linked with colon cancers^18^. We have reported a nonsense mutation in *PIK3R1* in an mRCC, while the mutation was absent in the corresponding primary renal cell carcinoma (pRCC)^14^. Therefore, we hypothesize that the downregulation of PIK3R1 may confer renal cancer cells a selective advantage to translocate, colonize and develop as mRCC.

We speculate that ectopic expression of PIK3R1 may be associated with progression and metastasis of RCC. To examine our hypothesis, we firstly analyzed the expression of PIK3R1 in RCC including both pRCC and mRCC by immunohistochemistry (IHC) and real-time polymerase chain reaction (RT-PCR). We discovered that the expression of PIK3R1 in RCC negatively correlated with tumor progression and metastasis. In addition, we induced deletion mutations of *PIK3R1* in renal cancer cell lines (786-O and A-704 cell lines) using a CRISPR/Cas9 system to achieve haploid knockout of *PIK3R1* which significantly decreased the expression of P85α. The mutated renal cancer cells displayed increased abilities of colony formation, tumor formation, migration, epithelial-mesenchymal transition and oncosphere formation. Thus, our current study demonstrates that the downregulation of PIK3R1 contributes to progression and metastasis of RCC.

## Results

### Downregulation of PIK3R1 correlates with progression and metastasis of RCC

**Figure 1:**
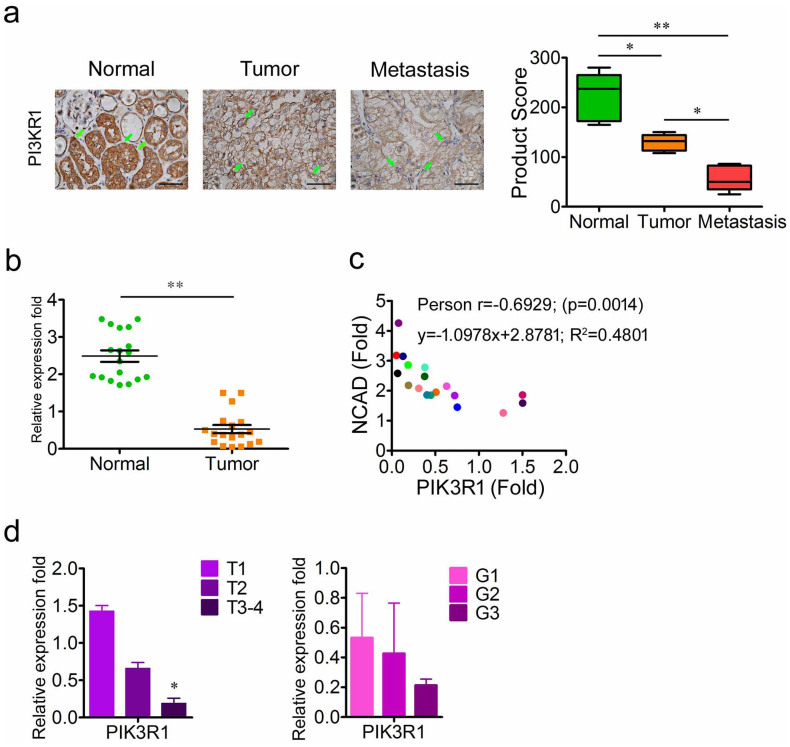
Downregulation of PIK3R1 correlates with progression and metastasis in RCCs. (a), The representative immunohistochemistry photographs of PIK3R1 expression in normal kidney tissues (n = 13), pRCCs (n = 13) and mRCCs (n = 21). Scale bar = 25 μm. (b), PIK3R1 mRNA levels in the pairs of pRCCs and corresponding normal renal tissues (n = 18). (c), PIK3R1 mRNA expression is negatively correlated with NCAD mRNA expression across a cohort of the pairs of normal renal tissues and pRCCs (n = 18). (d), PIK3R1 mRNA expression in pRCCs according to T category and grade. The TNM cancer staging system was designed to gauge the extent of cancer in a patient's body. T describes the size of the tumor and whether it has invaded nearby tissue, N describes regional lymph nodes that are involved, and M describes distant metastasis (spread of cancer from one body part to another). Grading classification for RCC was based on the Fuhrman grading system. Data are presented as mean ± SD. * *P* < 0.05, ** *P* < 0.01.

In order to examine the expression of PIK3R1 in RCC, the protein expression of PIK3R1 in normal kidney (n = 13), pRCC (n = 13) and mRCC (n = 21) was determined by IHC. As shown in Fig. 1a, normal kidney tissues displayed high level of PIK3R1 expression, whereas the expression of PIK3R1 was decreased in pRCC and was further reduced to a lower level in mRCC. The mRNA expression of PIK3R1 was then determined by real-time polymerase chain reaction (RT-PCR). Compared with normal kidney tissue group, the mRNA expression of PIK3R1 was significantly decreased in RCC group (n = 18) (Fig. 1b). The epithelial-mesenchymal transition (EMT) is considered to be crucial to tumor progression and metastasis, in which NCAD is the hallmark of EMT^19^,^20^. To determine whether the downregulation of PIK3R1 could affect the expression of NCAD, the mRNA expression of NCAD was examined, and data showed that the expression of NCAD had a negative correlation with the mRNA expression of PIK3R1 (Correlation = 0.6929, *P* = 0.0014) (Fig. 1c). Additionally, the mRNA expression of PIK3R1 negatively correlated with the T category of tumor, although there was no significant difference among different grades (Fig. 1d). These data suggest that the downregulation of PIK3R1 in RCCs correlates with their progression and metastasis.

### Haploid knockout of *PIK3R1* by CRISPR/Cas9 promotes tumor proliferation

**Figure 2:**
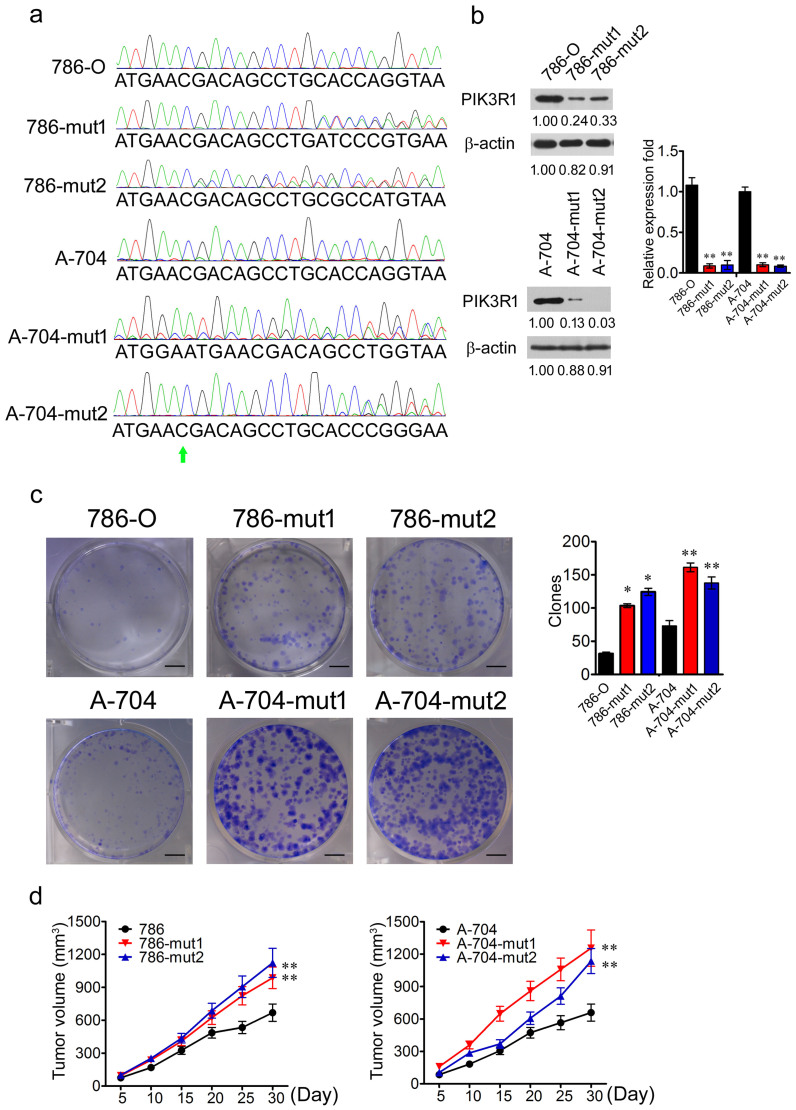
Haploid knockout of *PIK3R1* promotes tumor proliferation. (a), The Sanger sequencing of PCR product including chr5: 67576819C in 786-O, 786-mut1, 786-mut2, A-704, A-704-mut1, and A-704-mut2 cells. Arrowhead indicates chr5: 67576819C. (b), The RT-PCR and WB analysis of PIK3R1 expression in 786-O, 786-mut1, 786-mut2, A-704, A-704-mut1, and A-704-mut2 cells. β-actin served as control. (c), The colony formation assay of 786-O, 786-mut1, 786-mut2, A-704, A-704-mut1, and A-704-mut2 cells. Colonies were stained with crystal violet and measured after two weeks cultivation. (d), The tumor formation assays of 786-O, 786-mut1, 786-mut2, A-704, A-704-mut1, and A-704-mut2 cells. Scale bar = 50 mm. * *P* < 0.05, ** *P* < 0.01.

In order to reveal the functional influence of *PIK3R1* depletion, a CRISPR/Cas9 strategy which was designed according to the nonsense mutation reported previously^14^, was applied to induce a haploid deletion mutation in order to achieve haploid knockout of *PIK3R1*. Firstly, we analyzed the expression levels of PIK3R1 in normal renal cell line (HK2) and RCC cell lines (786-O, A-498, A-704, and ACHN) by using RT-PCR. 786-O and A-704 cell lines showed higher expression of PIK3R1 compared with that of HK2 cell line (Supplementary Fig. 1). Therefore, we performed depletion of *PIK3R1* in 786-O and A-704 cell lines. We also confirmed the sequence character of chr5: 67576819 in *PIK3R1* in 786-O and A-704 cell lines with Sanger sequencing. The 786-O and A-704 cell lines harbored the wild type (WT) sequence of chr5: 67576819C in *PIK3R1* (Fig. 2a).

We then applied the CRISPR/Cas9 technology^21^ to screen 786-O cells and A-704 cells with haploid deletion mutation of *PIK3R1*. After Sanger sequencing validation, we obtained muted 786-O cells (786-mut1 and 786-mut2) and A-704 cells (A-704-mut1 and A-704-mut2) with haploid deletion mutation (Fig. 2a). To determine the knockout efficiency in the 786 and A-704 mutated cells, RT-PCR and western blot (WB) were carried out to examine PIK3R1 expression in 786-O WT cells (786-O), mutated 786-O cells, A-704 WT cells (A-704), and mutated A-704 cells. The mRNA expression of PIK3R1 was significantly decreased in 786 and A-704 mutated cells compared with 786-O and A-704 WT cells (Fig. 2b). The protein levels of PIK3R1 were also significantly decreased in 786 and A-704 mutated cells respectively (*P* < 0.01; *P* < 0.01) (Fig. 2b).

To determine the effects of haploid knockout of *PIK3R1* on the growth of renal cancer cells, we carried out colony formation assays for the wild type and mutant cells. 786 and A-704 mutated cells displayed enhanced colony formation capability under the condition of separated single cells than that of 786-O and A-704 WT cells (Fig. 2c). In order to evaluate the tumor formation capability of the WT and muted cells, they were respectively inoculated subcutaneously into NOD/SCID mice to carry out tumor formation assays. The mutated cells formed bigger tumors than those of the wild type cells in NOD/SCID mice (Fig. 2d).

### Haploid knockout of *PIK3R1* promotes renal cancer cells migration and EMT *in vitro*

**Figure 3:**
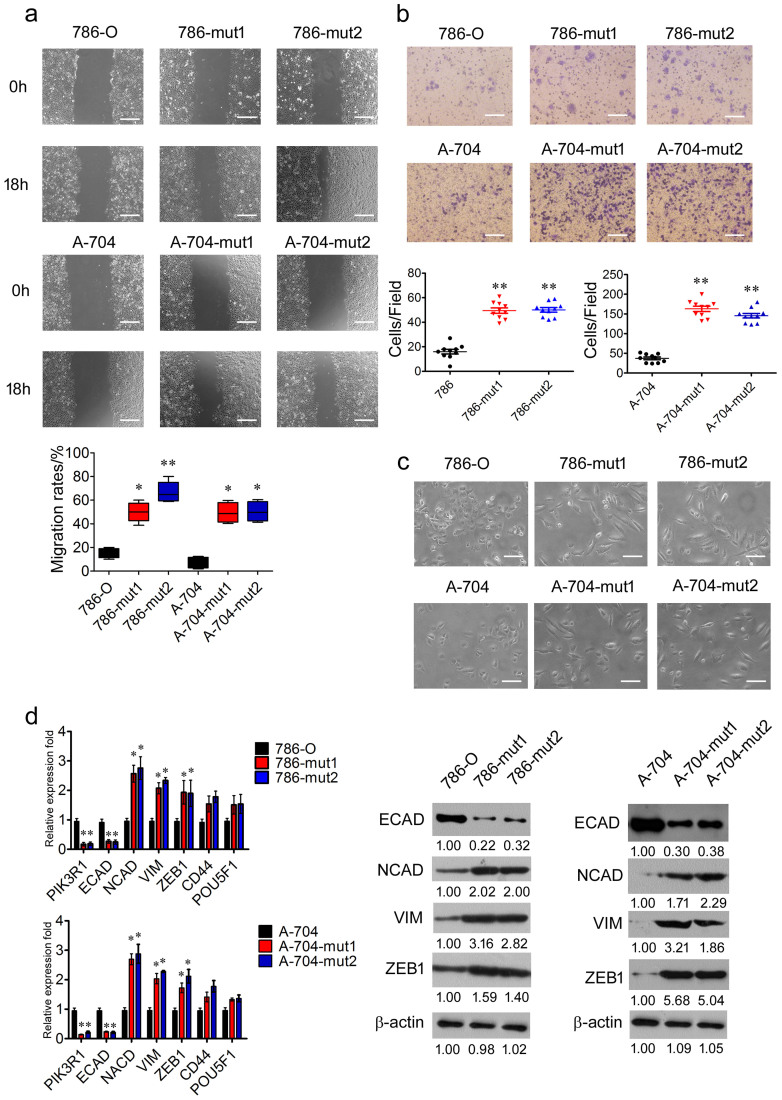
Haploid knockout of *PIK3R1* promotes migration and EMT in renal cancer cells. (a), The wound healing analysis of in 786-O, 786-mut1, 786-mut2, A-704, A-704-mut1, and A-704-mut2 cells. Migration rates were calculated by healing area/wound area. Scale bar = 50 μm. (b), Transwell of migration assays were applied to in 786-O, 786-mut1, 786-mut2, A-704, A-704-mut1, and A-704-mut2 cells. Scale bar = 25 μm. (c), Representative pictures of in 786-O, 786-mut1, 786-mut2, A-704, A-704-mut1, and A-704-mut2 cells. Scale bar = 25 μm. (d), The RT-PCR analysis of PIK3R1, ECAD, NCAD, VIM, ZEB1, CD44, and POU5F1 in 786-O, 786-mut1, 786-mut2, A-704, A-704-mut1, and A-704-mut2 cells. The WB analysis of ECAD, NCAD, VIM, and ZEB1 expression in 786-O, 786-mut1, 786-mut2, A-704, A-704-mut1, and A-704-mut2 cells. β-actin was applied as loading control. Data are expressed as mean ± SD. * *P* < 0.05, ** *P* < 0.01.

To confirm the effects of haploid knockout of *PIK3R1* on the migration of renal cancer cells, wound-healing and transwell assays for 786-O, 786-mut1, 786-mut2, A-704, A-704-mut1, and A-704-mut2 cells were carried out. Eighteen hours after artificial wounding, 786-O and A-704 WT cells demonstrated slight migration, whereas 786 and A-704 mutated cells showed enhanced migration ability and healed more than half of the wound (Fig. 3a). The 786 and A-704 mutated cells showed higher ability of migration in the transwell assay than that of 786-O and A-704 WT cells (Fig. 3b).

Additionally, 786 and A-704 mutated cells displayed a mesenchymal morphology^21^ that was different from the epithelial morphology of 786-O and A-704 WT cells, indicating that 786 and A-704 mutated cells might undergo EMT (Fig. 3c). In the wild type and mutant cells, the expression of EMT related gene was detected by RT-PCR and WB^20^. We found that, compared with 786-O and A-704 WT cells, in 786 and A-704 mutated cells with the downregulation of PIK3R1, the mRNA expression of ECAD was decreased, whereas the mRNA expression of NCAD, VIM and ZEB1 was increased (Fig. 3d). These observed changes were also found in the protein levels of ECAD (*P* < 0.01; *P* < 0.01), NCAD (*P* < 0.001; *P* < 0.05), VIM (*P* < 0.01; *P* < 0.05) and ZEB1 (*P* < 0.05; *P* < 0.05) in 786 and A-704 mutated cells respectively (Fig. 3d). However, no differences of CD44 and POU5F1 were observed between the wild type and mutant cells (Fig. 3d).

In order to confirm the function of PIK3R1 in renal cancer cells, we analyzed the expression of ECAD, NCAD, VIM, SNAIL, and TWIST in normal renal cell (HK2) and RCC cell lines (786-O, A498, A704, and ACHN) with RT-PCR. ACHN cell line showed higher expression of NCAD and VIM, compared to HK2 cell line (Supplementary Fig. 1). Therefore, we performed overexpression of PIK3R1 in ACHN cells. Compared with ACHN-vec cells, the mRNA expression of PIK3R1 and ECAD was increased in ACHN-PIK3R1 cells, whereas the expression of NCAD, VIM and ZEB1 was decreased (Supplementary Fig. 3a). These changes were also observed in the protein levels of PIK3R1 (*P* < 0.01), ECAD (*P* < 0.05), NCAD (*P* < 0.01), VIM (*P* < 0.05), and ZEB1 (*P* < 0.05) in ACHN-PIK3R1 cells (Supplementary Fig. 3b). In addition, ACHN-PIK3R1 cells displayed a epithelial morphology^21^ that was different from the mesenchymal morphology of ACHN-vec cells (Supplementary Fig. 3c). These results indicated that ACHN-PIK3R1 cells might undergo mesenchymal-epithelial transition (MET). Taken together, *PIK3R1* negatively regulated renal cancer cell migration and EMT in vitro.

### Haploid knockout of *PIK3R1* promotes a cancer stem cell phenotype

**Figure 4:**
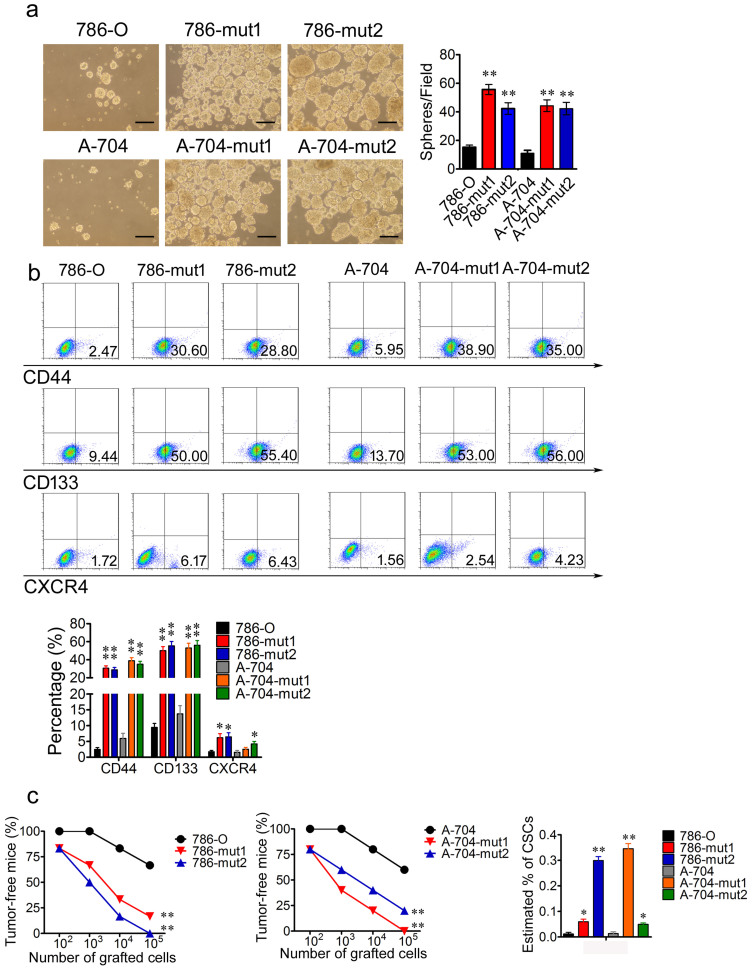
Haploid knockout of *PIK3R1* promotes cancer stem cell phenotype through activating *CTNNB1*. (a), The representative photographs of cell spheres formed by 786-O, 786-mut1, 786-mut2, A-704, A-704-mut1, and A-704-mut2 cells. Spheres were calculated after two weeks cultivation at five independent fields/well. Scale bar = 50 μm. (b), 786-O and A-704 WT cells comprise fewer CD44^+^, CD133^+^, and CXCR4^+^ subset, respectively, compared with 786-O and A-704 WT cells. (c), The percentage of tumor-free mice 1 month after the subcutaneous injection of the different dilutions of 786-O, 786-mut1, 786-mut2, A-704, A-704-mut1, and A-704-mut2 cells into immunodeficient mice. The estimated percentage of renal cancer stem cells (RCSCs) in 786-O WT, 786-O Mut, A-704 WT, and A-704 Mut after the first transplantation using the extreme limiting dilution analysis. * *P* < 0.05, ** *P* < 0.01.

Cancer stem cells (CSCs) are defined as a subpopulation in tumors which harbors selfrenew, differentiation and serial tumor formation ability^22^. Cancer cell lines contain cancer stem-like cells which can form cell spheres under sphere-forming conditions^23^. To evaluate whether the reduction of PIK3R1 influences the cancer stem cell phenotype of renal cancer cells, cell sphere formation assays of 786-O, 786-mut1, 786-mut2, A-704, A-704-mut1, and A-704-mut2 cells were performed. After two weeks of cultivation, 786 and A-704 mutated cells developed more and bigger spheres than that of 786-O and A-704 WT cells under the medium supplemented with 20 ng/mL EGF, FGF, N2, and B27 (Fig. 4a). CSCs have been identified based on expression of various markers such as CD44, CD133 and CXCR4^24^,^25^,^26^. By using anti-CD44 antibodies, anti-CD133 antibodies and anti-CXCR4 antibodies we observed that 786 and A-704 mutated cells comprised more CD44^+^, CD133^+^ and CXCR4^+^ subset, respectively, compared with 786-O and A-704 WT cells (Fig. 4b).

The gold standard assay to assess CSCs potential is the transplantation of limiting dilutions of highly purified prospectively identified cancer cell populations into immunodeficient mice to assess their ability to form tumors^27^,^28^. Transplantation of different dilutions of the wild type cells and *PIK3R1* mutated cells showed that the mutated cells had a much greater ability to initiate tumors and increasing percentage of CSCs compared with the WT cells (Fig. 4c).

### Haploid knockout of *PIK3R1* activates WNT/β-catenin pathway dependent on the phosphorylation of AKT

**Figure 5:**
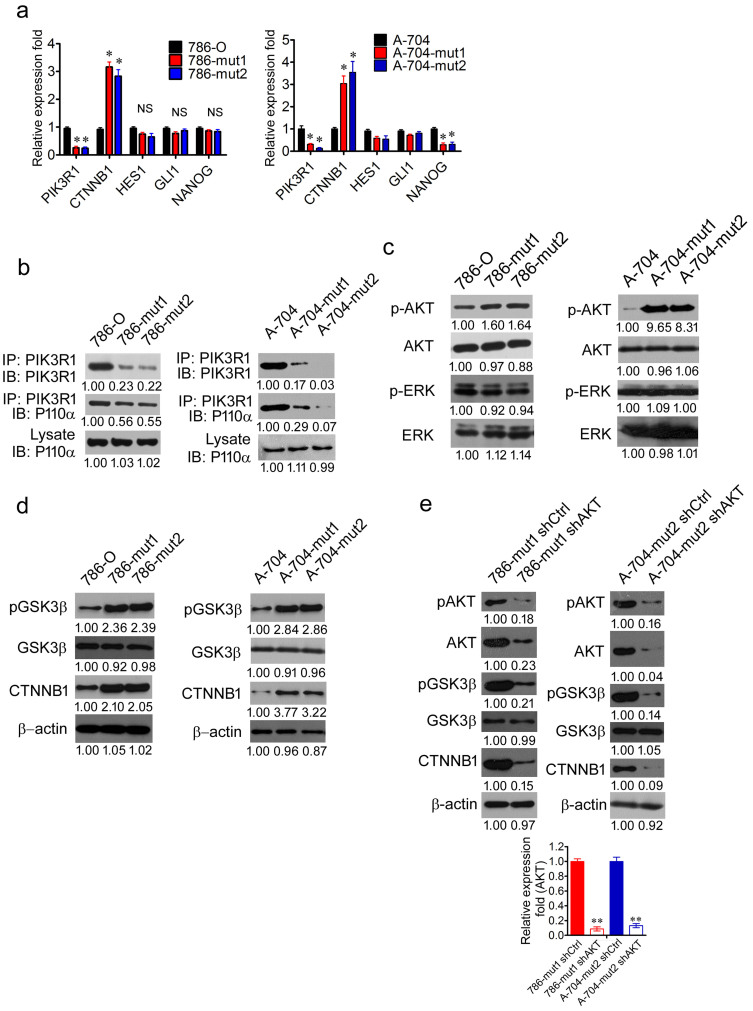
Haploid knockout of *PIK3R1* activates WNT/β-catenin pathway dependent on the phosphorylation of AKT. (a), The RT-PCR analysis of the mRNA expression of PIK3R1, CTNNB1, HES1, GLI1, and NANOG in 786-O, 786-mut1, 786-mut2, A-704, A-704-mut1, and A-704-mut2 cells. (b), 786-O, 786-mut1, 786-mut2, A-704, A-704-mut1, and A-704-mut2 cell lysates were immunoprecipitated by anti-PIK3R1 antibody and immunoblotted with anti-PIK3R1 (upper panel) and anti-P110 antibody (middle panel). Whole lysates immunobotted with anti-P110 antibody (lower panel) served as control. (c), The WB analysis of p-AKT and p-ERK in 786-O, 786-mut1, 786-mut2, A-704, A-704-mut1, and A-704-mut2 cells. AKT and ERK served as control. (d), The WB analysis of CTNNB1, p-GSK3β, GSK3β in 786-O, 786-mut1, 786-mut2, A-704, A-704-mut1, and A-704-mut2 cells. (e), The WB analysis of AKT, pAKT, pGSK3β, GSK3β, and CTNNB1 in 786-mut1 shCtrl, 786-mut1 shAKT, A-704-mut2 sh Ctrl, and A-704-mut2 shAKT cells. The RT-PCR analysis of AKT in 786-mut1 shCtrl, 786-mut1 shAKT, A-704-mut2 shCtrl, and A-704-mut2 shAKT cells. Data are presented as mean ± SD. * *P* < 0.05, ** *P* < 0.01.

In order to uncover the stem cell signaling pathway activated by the downregulation of PIK3R1, the mRNA level of CTNNB1, HES1, GLI1, and NANOG were analyzed in 786-O, 786-mut1, 786-mut2, A-704, A-704-mut1, and A-704-mut2 cells by RT-PCR. With the downregulation of PIK3R1 in 786 and A-704 mutated cells, the mRNA expression of *CTNNB1* was higher than that in 786-O and A-704 WT cells (Fig. 5a). The protein levels of *CTNNB1* were also increased in 786 and A-704 mutated cells respectively (*P* < 0.01; *P* < 0.05) (Fig. 5d) There were no significant differences in the mRNA expression of HES1 and GLI1 between the wild type and mutated cells (Fig. 5a). Though there were no significant differences in the NANOG mRNA expression between 786-O WT and 786 mutated cells, significant difference was observed between A-704 WT and A-704 mutated cells (Fig. 5a).

In order to determine the molecular mechanism underlying β-catenin (*CTNNB1*) activation mediated by *PIK3R1* depletion, immunoprecipitation with PIK3R1 antibody was carried out by using lysates of 786-O, 786-mut1, 786-mut2, A-704, A-704-mut1, and A-704-mut2 cells. The quantity of p110α immunoprecipitated by PIK3R1 was decreased due to knockout of *PIK3R1* in 786 and A-704 mutated cells respectively, compared with 786-O and A-704 WT cells (*P* < 0.05; *P* < 0.05) (Fig. 5b). Additionally, more AKT was phosphorylated in 786 and A-704 mutated cells (*P* < 0.05; *P* < 0.01), compared with 786-O and A-704 WT cells that had the amount of unchanged AKT (*P* = 0.54; *P* = 0.49) (Fig. 5c). However, there was no significant change in the quantity of ERK (*P* = 0.07; *P* = 0.45) and ERK with phosphorylation (*P* = 0.08; *P* = 0.30) in 786 and A-704 mutated cells respectively (Fig. 5c).

He, K. *et al* reported that cancer cells acquired cancer stem-like phenotype through alteration of the PI3K/AKT/β-catenin signaling^29^. According to a previous study^30^, the phosphorylation of AKT could activate the WNT/β-catenin pathway through phosphorylating and inhibiting GSK3β. Interestingly, more GSK3β was phosphorylated in 786 and A-704 mutated cells compared with 786-O and A-704 WT cells (*P* < 0.01; *P* < 0.001) (Fig. 5d). To determine the function of AKT in the mutated cells, depletion of AKT were performed in 786-mut1 and A-704-mut2 cells. After the depletion of AKT (*P* < 0.001; *P* < 0.001), the expression of pAKT (*P* < 0.01; *P* < 0.001), p-GSK3β (*P* < 0.001; *P* < 0.001) and CTNNB1 (*P* < 0.001; *P* < 0.05) was decreased in 786-mut1 shAKT and A-704-mut2 shAKT cells respectively, compared with 786-mut1 shCtrl and A-704-mut2 sh Ctrl cells (Fig. 5e). Taken together, the depletion of PIK3R1 activates the WNT/β-catenin pathway dependent on the phosphorylation of AKT.

### Haploid knockout of *PIK3R1* promotes a cancer stem cell phenotype through WNT/β-catenin pathway

**Figure 6:**
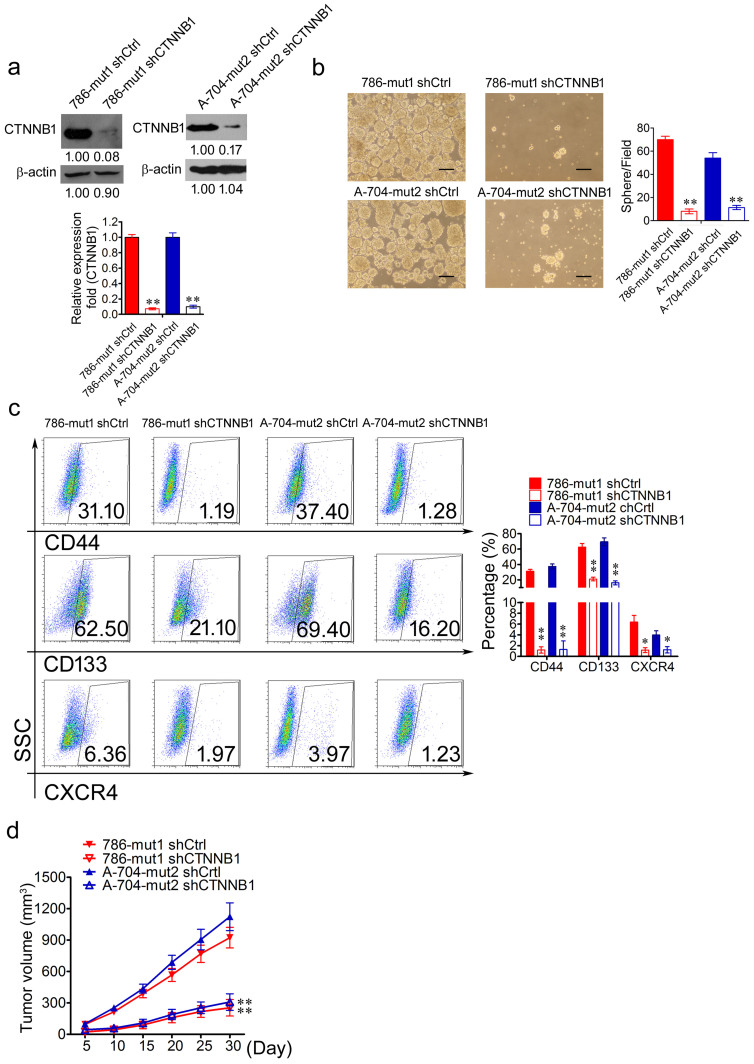
Haploid knockout of *PIK3R1* promotes cancer stem cell phenotype through activating *CTNNB1*. (a), The WB and RT-PCR analysis of CTNNB1 in 786-mut1 shCtr, 786-mut1 shCTNNB1, A-704-mut2 shCtr, and A-704-mut2 shCTNNB1 cells. (b), The representative photographs of cell spheres formed by 786-mut1 shCtr, 786-mut1 shCTNNB1, A-704-mut2 shCtr, and A-704-mut2 shCTNNB1 cells. Scale bar = 50 μm. (c), 786-mut1 shCTNNB1 and A-704-mut2 shCTNNB1 cells comprised fewer CD44^+^, CD133^+^ and CXCR4^+^ subset, respectively, compared with 786-mut1 shCtr and A-704-mut2 shCtr cells. (d), The tumor formation assays of 786-mut1 shCtrl, 786-mut1 shCTNNB1, A-704-mut2 shCtrl, and A-704-mut2 shCTNNB1 cells. * *P* < 0.05, ** *P* < 0.01.

To determine the function of *CTNNB1* in the mutated cells, depletion of *CTNNB1* was performed in 786-mut1 and A-704-mut2 cells respectively. The mRNA expression of *CTNNB1* was decreased in 786-mut1 shCTNNB1 and A-704-mut2 shCTNNB1 cells, compared to 786-mut1 and A-704-mut2 cells (Fig. 6a). The decrease of *CTNNB1* protein was also observed in 786-mut1 shCTNNB1 and A-704-mut2 shCTNNB1 cells (*P* < 0.01; *P* < 0.01) (Fig. 6a). After two weeks of cultivation, 786-mut1 shCTNNB1 and A-704-mut2 shCTNNB1 cells developed fewer and smaller spheres than that of 786-mut1 shCtrl and A-704-mut2 shCtrl cells (Fig. 6b). By using anti-CD44 antibodies, anti-CD133 antibodies and anti-CXCR4 antibodies, we observed that 786-mut1 shCTNNB1 and A-704-mut2 shCTNNB1 cells comprised fewer CD44^+^, CD133^+^ and CXCR4^+^ subset, respectively, compared with 786-mut1 shCtrl and A-704-mut2 shCtrl cells (Fig. 6c). In order to evaluate the tumor formation capability of 786-mut1 shCTNNB1, A-704-mut2 shCTNNB1, 786-mut1 shCtrl, and A-704-mut2 shCtrl cells, they were respectively inoculated subcutaneously into NOD/SCID mice to carry out tumor formation assays. 786-mut1 shCTNNB1 and A-704-mut2 shCTNNB1 cells formed smaller tumors than those of 786-mut1 shCtrl and A-704-mut2 shCtrl cells in NOD/SCID mice (Fig. 6d). These results indicated that the haploid knockout of *PIK3R1* enhanced the abilities of self-renewal and tumor formation dependent on the activation of the PI3K/AKT/CTNNB1 pathway in renal cancer cells.

## Discussion

Over the past 20 years, the functional role of the PI3K/AKT pathway in tumorigenesis has been reported by numerous studies^31^. Members of the pathway such as *PIK3CA*, *AKT* and *PTEN*, are frequently altered, and cause the activation of the pathway in RCC^2^,^14^. As a regulatory subunit of PI3Ks, PIK3R1 alteration is involved in carcinogenesis in a variety of cancers^9^, whereas the alteration of PIK3R1 in RCC has been rarely reported^14^. In this study, we report that *PIK3R1* expression is reduced in RCC, and that the reduction of PIK3R1 can result in EMT, enhance migration ability, and promote colony formation, tumor formation and cancer stem cell phenotype in renal cancer cells.

Our data showed that PIK3R1 expression was reduced in RCC, especially in advanced and metastatic RCC, and the downregulation of PIK3R1 correlated with advanced or metastatic RCC. These results are in consistence with discoveries in hepatocellular and colon cancers^16^,^18^, indicating that the reduction of PIK3R1 expression may acquired tumorigenicity in RCC, and thus supporting the view that *PIK3R1* may function as a potential cancer suppressor, and that the downregulation of PIK3R1 may promote progression and metastasis of RCC.

We then tested the role of *PIK3R1* in renal cancer cell lines. We observed that the haploid deletion mutations of *PIK3R1* by CRISPR/Cas9 system led to the haploid knockout and downregulation of PIK3R1, and generated cell lines with the insufficiency of PIK3R1. The binding of p110α to p85α was decreased in 786 and A704 mutated cells with the reduced expression of PIK3R1. We also showed that the downregulation of PIK3R1 functionally activated the PI3K/AKT pathway, as evidenced by the enhanced phosphorylation of AKT in Ser473 residue. The activation of the pathway may be due to the combined effect of several factors. The p85 regulatory subunit of PI3K is necessary to stabilize and recruit p110 catalytic subunit onto cellar membrane^13^. However, monomeric p85 is in competition with p85-p110 heterodimers for binding to insulin receptor substrate-1 (IRS-1) to form a non-signaling cytosolic protein complex^32^. The insufficiency of PIK3R1 expression would primarily result in the reduction of monomeric p85, which would suppress the negative regulation of IRS-1/PI3K pathway by p85, thus leading to the hyperactivation of PI3K signaling axis^32^. *PIK3R1* has also been shown to have a positive regulatory effect on tumor suppressor *PTEN*, which negatively regulates the PI3K/AKT pathway^16^. Therefore, the downregulation of PIK3R1 leads to the inhibition of *PTEN* function that reduces the breakdown of PIP3, and activates the downstream of the PI3K/AKT signaling axis^16^.

Our data show that the reduction of PIK3R1 expression increases the motility and migration capacity of renal cancer cells. A growing body of evidence indicates that the PI3K/AKT pathway is involved in the migratory process of cells, including metastatic cancer cells^33^. Activated AKT mediates cell polarity and reorganizes cytoskeleton, thus regulates the contraction of the cellular body and facilitates the migration of cells^33^. AKT-mediated phosphorylation activates components closely related to the cellular filaments which are important for cytoskeleton dynamics^34^. In our present study, the expression of VIM was elated in cells with the downregulation of PIK3R1. Vimentin, which is phosphorylated and protected by AKT from degradation^35^, is substantially up-regulated when cells are highly motile, especially during EMT. It can promote the migration of different type of cells such as cancer cells^35^.

Interestingly, spindle-shaped morphology of mesenchymal cells has been observed in cells with the down-expression of PIK3R1. EMT is conducive to the survival and spread of cancer cells^36^. The activation of AKT induces EMT and contributes to migration of squamous cell carcinoma lines^37^. Evidences are emerging that the action of AKT is related with EMT-inducing transcription factor^38^. The hallmarks of EMT include decreased expression of epithelial marker, such as ECAD, and a simultaneous increased expression of mesenchymal markers, such as NCAD, VIM, CD44, ZEB1, and POU5F1^20^. In our study, the expression of NCAD, VIM and ZEB1 was elated in the cells with the downregulation of PIK3R1, whereas the expression of ECAD was decreased in these cells. The activated AKT positively regulates the expression of NCAD^33^, and the inhibition of the PI3K/AKT signaling inhibits the expression of NCAD^39^. N-cadherin encoded by *NCAD* is enriched in cellular protrusions and plays a key role in cell migration^33^. Altogether, through the activation of the PI3K/AKT pathway which phosphorylates several cytoskeleton-regulating and EMT–activating proteins, the downregulation of PIK3R1 promotes migration and EMT in renal cancer cells.

CSCs are defined as cells which are able to produce a new tumor^22^, and regarded as a source of tumor progression and metastasis^22^. Cancer cell lines contain cancer stem-like cells^23^. In our current study, we observed that the down-expression of PIK3R1 promoted the colony formation, cell sphere and tumor formation of renal cancer cells and increased the expression of *CTNNB1*. Our data are consistent with the study by Zhou *et al,* who reported that the CSC viability and maintenance of breast cancer stem-like cells required the activation of the PI3K/AKT signaling axis^40^. It has been suggested that the PI3K/AKT pathway is critical to maintain prostate cancers stem-like cells^41^. Signaling pathways including the Hedgehog/GLI1, WNT/CTNNB1, NANOG/OCT4, and Notch/HES1 are activated in CSCs and contribute to the function of CSCs^42^. We performed RT-PCR to reckon the expression of CTNNB1, HES1, GLI1, and NANOG. Interestingly, no significant change was observed in the expression of HES1, GLI1 and NANOG, whereas the expression of CTNNB1 was markedly increased, suggesting that CTNNB1 may be involved in increased renal cancer stem-like phenotype caused by the down-expression of PIK3R1.

Our present study demonstrates that the downregulation of PIK3R1 in RCC enhances stem cells expansion as a result of the activated PI3K/AKT signaling, involving the WNT/CTNNB1 pathway. The PI3K/AKT pathway has always been ascribed to the proliferation and self-renewal of cancer stem-like cells^29^. However, under certain conditions, such as ectopic gene expression, cancer stem-like cells can also be transformed from cells lacking cancer stem-like phenotype^43^. The activation of the PI3K/AKT pathway has been reported to induce differentiated cells to transform into highly tumorigenic and cancer stem-like cells^44^. In our study, after the depletion of AKT, the expression of p-GSK3β and CTNNB1 was decreased in 786-mut1 shAKT and A-704-mut2 shAKT cells. Therefore, in consistence with previous studies, our study suggests that the PI3K-AKT and WNT/CTNNB1 pathways converge on GSK3β in renal cancer cells^29^. The WNT/CTNNB1 signaling is involved in regulating the various types of stem cells^29^. In present study, the depletion of *CTNNB1* in renal cancer cells decreased the cancer stem-like phenotype of these cells. Activated AKT can block the kinase activity of GSK3β by phosphorylating GSK3β^30^, resulting in the accumulation of β-catenin which transforms differentiated cancer cells to cancer stem-like cells^29^,^30^. Therefore, the PI3K/AKT and WNT/CTNNB1 pathway work together as a PI3K/AKT/GSK3β/CTNNB1 pathway which induces differentiated renal cancer cells to transform into a more cancer stem-like cells^29^.

To our knowledge, this study is the first report of the functional identification of *PIK3R1* in RCC. The important discovery of our study is that *PIK3R1* is down expressed in RCC, especially in advanced and metastatic RCC. We observed that the downregulation of PIK3R1 resulted in EMT, increased migration, colony formation, cell sphere, and tumor formation in renal cancer cells, suggesting that the reduction of PIK3R1 expression confers renal cancer cells a selective advantage to translocate, colonize and develop as mRCC, and that *PIK3R1* may be involved in the progression and metastasis of RCC through the PI3K/AKT/GSK3β/CTNNB1 pathway. Since the reduction of PIK3R1 expression and activating alterations in *PIK3CA* can simultaneously be involved in RCC, conformational changes may result from these alterations. These changes would eliminate the repression of *PIK3CA* caused by *PIK3R1*^32^. Therefore, the exploitation of agents that can restore the repression regulation of PIK3R1 and inhibit the expression of PIK3CA, may be a new strategy to treat RCC.

In summary, our study suggests that PIK3R1 is down-regulated in RCC, especially in advanced and metastatic RCC. The downregulation of PIK3R1 promotes EMT, migration, and cancer stem cell phenotype in renal cancer cells and may contribute to the progression and metastasis of RCC by activating the PI3K/AKT/GSK3β/CTNNB1 pathway. Increasing or restoring expression of PIK3R1 may be a novel treatment strategy for RCC.

## Methods

### Reagent and antibody

Antibodies that recognize pAKT (Ser473) (Cell Signaling Technology, #9271), AKT (Cell Signaling Technology, #9272), pERK (Thr202/Tyr204) (Cell Signaling Technology, #4370), ERK (Cell Signaling Technology, #4695), p85α (Santa Cruz, sc-376112), p110α (Cell Signaling Technology, #4249), β-actin (Sigma, A1978), NCAD (Abcam, AB98952), ECAD (Cell Signaling Technology, #3195), VIM (RV202) (Abcam, AB8978), ZEB1 (D80D3) (Cell Signaling Technology, #3396), pGSK3β (Ser9) (Cell Signaling Technology, #9323), GSK3β (Y174) (Abcam, AB32391), and non-phospho CTNNB1 (Ser33/37/Thr41) (Cell Signaling Technology, #4270) were used in the study. Horseradish peroxidase (HRP) labelled secondary antibody (Beyotime Biotech) was used in western blots. Protein A-Sepharose 4B beads (Invitrogen, 10-1041) was used for immunoprecipitation studies.

### Patients and tissue specimens

For the protein expression of PIK3R1, the formalin-fixed, paraffin-embedded (FFPE) tissue samples of patient-matched normal renal, pRCC and mRCC tissues from 13 patients who underwent nephrectomy between May 2008 and April 2013 were obtained from the archives of the Zhujiang Hospital of the Southern Medical University (Supplementary table 1). The biopsy samples of mRCCs from eight patients who underwent biopsies of metastases but not nephrectomy between May 2009 and September 2013 were also collected in the hospital (Supplementary table 1). Median age of these patients was 56 years (range: 16–71 years). Thirteen of patients were male and the rest were female. For the mRNA expression of identified genes, pRCCs tissues and corresponding normal renal tissues from 18 patients who underwent nephrectomy in the hospital between August 2012 and October 2013 were immediately collected following surgical resection (Supplementary table 2). The median age of these patients was 50 years (range: 29–65 years). Thirteen of these patients were male and the others were female. The disease stage of all patients was classified according to the American Joint Committee on Cancer staging system^45^. This study was reviewed and approved by the Ethics Committee of the Southern medical University, and was carried out according to the World Medical Association Declaration of Helsinki. The written informed consents were obtained from all patients or their guardians.

### Immunohistochemistry

Sections (4 μm) were deparaffinized and rehydrated. After antigen retrieval, these sections were treated with 3% H_2_O_2_ solution, incubated with 8% bovine serum albumin for 30 min and primary antibody at 4°C overnight, then incubated with corresponding secondary antibody, and subsequently stained with DAB kit (ZSGB Bio). The nucleus was counterstained with hematoxylin. The overall staining for PIK3R1 was measured by the multiplication of staining percentage (0%–100%) and staining intensity on a numerical scale (none = 1, weak = 2, moderate = 3, strong = 4), resulting in an overall product score.

### Real-Time PCR analysis of *PIK3R1* expression

Total RNA was extracted using RNA isolation kit (Tiangen Biotech) and subjected to cDNA synthesis using PrimeScript RT reagent Kit (Takara Bio). The cDNA was then used for the evaluation of the relative mRNA levels of the indentified gene, running in an ABI 7300 analyzer (Applied Biosystems). SYBR Green I (Tiangen Biotech) was used as the fluorescent probe. Primer sequences for RT-PCR are listed in the supplementary table 3. The relative expression levels of the target genes were referred to as a housekeeping gene, GAPDH.

### Cell line

HK-2 cells were cultured in DMEM/F12 medium (Gibco). Other cells were maintained in RPMI 1640 medium (Gibco). All mediums were supplemented with 10% fetal bovine serum (FBS) (Gibco), 100 U/mL penicillin (Gibco) and 100 mg/mL streptomycin (Gibco), and incubated at 37°C with 5% CO_2_ in a humidified atmosphere.

### *PIK3R1* knockout by CRISPR/Cas9

The knockout of *PIK3R1* in 786-O and A-704 cell lines was achieved by CRISPR/Case9 system. One 23-base sgRNA (5′-AATGAACGACAGCCTGCACCAGG-3′) was designed to the target site (g.chr5: 67576819) in the *PIK3R1* (Gene ID: 5295). The U6-gRNA expression cassette including U6 promoter and +83 bp sgRNA tails was synthesized (Invitrogen) and inserted into pCEP4 vector (Invitrogen) by KpnI and XhoI. The sgRNA was synthesized (Invitrogen) and inserted into two AarI site between U6 promoter and sgRNA tails to form the pCEP4-U6-gRNA expression vector (Supplementary Figure 2). The sequence of Cas9 was consistent with previous reports^46^,^47^. The T7 promoter was added upstream of the Cas9 sequence. All the sequences were synthesized (Invitrogen) and ligated to the pMD-18T-CMV-MCS-BGPA by NheI/AflII to form CMV-Cas9 expression vector (Supplementary Figure 2).

786-O and A-704 cells were transfected according to Lipo-fectamine 2000 (Invitrogen) protocol. Briefly, Cells were transfected at 60% confluency in 12 well with 500 ng of pCEP4-U6-gRNA expression vector, 500 ng of CMV-Cas9 expression vector and 3 μL Lipo-fectamine 2000. After G418 (500 ng/μL) selection for 10 days, G418-resistant colonies were picked and cultured in 24 well plates. Genomic DNA was extracted with E. Z.N.A. Tissue DNA Kit (OMEGA). Targeted cleavage was measured by PCR amplification (forward primer: 5′ CTCATCAGTATTGGCTTACGCTT 3′ and reverse primer: 5′ GGTTGTTTAGACTTTCCACGGTA 3′) and T7 E1 assay. The fragments were determined by Sanger sequencing. To better verify the mutations (insertions and deletions), the PCR product of mutated colonies was then ligated to pMD-18T vector (Takara Bio). Individual mutation and mutational spectra were obtained from Sanger sequence. Cell clones with haploid deletion mutation of *PIK3R1* were selected for evaluating the function of *PIK3R1* in renal cancer cells.

### Western blot and immunoprecipitation (IP)

Cells were lysed with radioimmunoprecipitation assay buffer (Beyotime Biotech). Protein was determined, separated and transferred to polyvinylidene difluoride membranes (Pall Life Sciences). The membranes were blocked, then probed with corresponding primary antibody and HRP-conjugated secondary antibodies (Beyotime Biotech), and detected using BeyoECL PLUS Kit (Beyotime Biotech).

For immunoprecipitation assays, cell lysates were incubated with the PIK3R1 antibody overnight and precipitated with protein A-Sepharose 4B beads (Invitrogen) at 4°C for 4 h. The immunoprecipitated proteins were washed, separated on SDS-PAGE, transferred onto a PVDF membrane, and followed by western blot analysis.

### Colony formation assay

Briefly, single cells were seeded onto 6-well plates (Corning) at a density of 1000 cells/well and then incubated at 37°C. After two weeks of culture, colonies were stained with 0.5% crystal violet solution for 30 min at room temperature. The colonies from three replicate wells were counted, and pictures were captured digitally.

### Generation of xenografts

For generation of xenografts, different dilutions of cells were injected with Matrigel (BD Biosciences) subcutaneously into the backs of NOD/SCID mice. The volume of xenografts was serially measured. The mice were sacrificed after 30 days. Animal work was carried out in compliance with the ethical regulations approved by the Animal Care Committee, Southern medical University.

### Wound healing assay

Briefly, 4 × 10^5^ cells were seeded into a 24 well plate. After overnight incubation, monolayer cells grew to confluency. Artificial wounds were introduced with the P200 pipette tip. The pictures of the wounded area were taken immediately (time 0 h), and at 18 h with an inverted microscope (Olympus Corp). The whole assay was repeated three times.

### Transwell Assay

Cells were respectively harvested, suspended and added to the upper compartment of transwell inserts (8 μm pore size; Corning). The lower chambers were filled with RPMI-1640 supplemented with 10% FBS. After incubation for 16 hr, cells that had not migrated from the upper chamber were scraped away. The cells that migrated to the lower surface of each membrane were stained and counted under a light microscope in in five fields/well. The whole assay was repeated three times.

### Spheres Culture

Single cells were seeded at a density of 5 × 10^3^ cells/well in 6-well plates with ultra-low attachment surface (Corning). Cells were maintained in serum-free 1:1 DMEM/F12 (Gibco) supplemented with 20 ng/mL EGF (Invitrogen), 20 ng/mL bFGF (Invitrogen), 2% B27 (Invitrogen), 1% N2 (Invitrogen), and 100 mg/mL streptomycin (Gibco). The number of tumor spheres was counted 14 days after seeding. The whole assay was repeated three times.

### Cell labeling and flow cytometry analyzing

Cells were harvested and washed twice in ice-cold PBS. Then cancer cells were stained with FITC-conjugated anti-CD133 antibody, PE-conjugated anti-CD44 antibody and FITC-conjugated anti-CXCR4 antibody, respectively, for 30 min on ice. After washing with PBS, the labeled cells were analyzed by flow cytometry (BD FACSAria III).

### Knockdown of *AKT and CTNNB1* with shRNA

Four shRNA targeted against the different regions of *AKT* and *CTNNB1* and scrambled shRNAs were designed, synthesized and cloned into pSicoR vector: ShCTNNB1: Ccgg CAGATGGTGTCTGCTATTGTACTCGAGTACAATAGCAGACACCATCTGTTTTT g; shCtrl: CcggTTCTCCGAACGTGTCACGTCTCGAGACGTGACACGTTCGGAGA ATTTTTg; shAKT: CcggCCTCAAGAATGATGGCACCCTCGAGGGTGCCATCAT TCTTGAGGTTTTTg; shAKT Ctrl: CcggAGTTCCAGTACGGCTCCAACTCGAGTT GGAGCCGTACTGGAACT TTTTTg. The resulting lentiviral vectors containing the CTNNB1 and AKT shRNA were named shCTNNB1 and shAKT lentivirus. Cells were transfected with the shCTNNB1 and shAKT lentivirus to obtain cell lines stably expressing the CTNNB1 and AKT shRNA.

### Overexpression of *PIK3R1*

*PIK3R1* (NM_181523.2) was amplified by PCR with the following primers (forward: 5'- CGG AAT TCC ATG CAT GGT GAT TAT ACT CTT ACA -3' and reverse: 5'- CGC GGA TCC TCA TCG CCT CTG CTG TGC ATA TAC -3'). The PCR product was purified and cloned into pSin-GFP-vector to obtain Psin-GFP-PIK3R1. ACHN cells were transfected with the pSin-GFP-PIK3R1 lentivirus to obtain a cell line stably expressing the *PIK3R1* cDNA.

### Statistical analysis

Student's t-test was used to comparing the means of two samples. Linear correlation analysis was used to determine relationship between PIK3R1 and NCAD mRNA expression. A value of p less than 0.05 (* *P* < 0.05, ** *P* < 0.01) was regarded statistically significant.

## Author Contributions

Y.L., Z.Y., H.L. (Huan Liu), F.L., D.S., X.Z. and X.W. performed the experiments and analyzed the data. C.L. and S.W. designed the project and wrote the main manuscript text. A.X., P.D., Y.H., D.Z., H.L. (Hongjie Liu), M.Z. and Y.F. edited various parts of the manuscript text. A.X., H.W., Q.X., Y.W. (Yongqiang Wang), Y.Z., Y.W. (Yu Wang) and Z.C. helped with the experimental design. C.L. and S.W. supervised the data analysis and edited text for the manuscript. All authors reviewed the manuscript.

## Supplementary Material

Supplementary Information Supplementary data

## Notes

**Financial Support:** This project was supported by theNational Natural Science Foundation of China (Grant No. 81301740) and Innovation Program of Shenzhen (Grant No. JCYJ20130401114715714; No. JCYJ20130401114928183; No. JSGG20130411091246833; No. CXZZ2013051653248144).

## References

[b1] Rini B. I., Campbell S. C. & Escudier B. Renal cell carcinoma. Lancet 373, 1119–32 (2009). 19269025 10.1016/S0140-6736(09)60229-4

[b2] Comprehensive molecular characterization of clear cell renal cell carcinoma. Nature 499, 43–9 (2013). 23792563 10.1038/nature12222 PMC3771322

[b3] Guo G. *et al.* Frequent mutations of genes encoding ubiquitin-mediated proteolysis pathway components in clear cell renal cell carcinoma. Nat Genet 44, 17–9 (2012). 22138691 10.1038/ng.1014

[b4] Varela I. *et al.* Exome sequencing identifies frequent mutation of the SWI/SNF complex gene PBRM1 in renal carcinoma. Nature 469, 539–42 (2011). 21248752 10.1038/nature09639 PMC3030920

[b5] Ather M. H., Masood N. & Siddiqui T. Current management of advanced and metastatic renal cell carcinoma. Urol J 7, 1–9 (2010). 20209445

[b6] Porta C. & Figlin R. A. Phosphatidylinositol-3-kinase/Akt signaling pathway and kidney cancer, and the therapeutic potential of phosphatidylinositol-3-kinase/Akt inhibitors. J Urol 182, 2569–77 (2009). 19836781 10.1016/j.juro.2009.08.085

[b7] Yan W. *et al.* PI3 kinase/Akt signaling mediates epithelial-mesenchymal transition in hypoxic hepatocellular carcinoma cells. Biochem Biophys Res Commun 382, 631–6 (2009). 19303863 10.1016/j.bbrc.2009.03.088

[b8] Miller T. W., Rexer B. N., Garrett J. T. & Arteaga C. L. Mutations in the phosphatidylinositol 3-kinase pathway: role in tumor progression and therapeutic implications in breast cancer. Breast Cancer Res 13, 224 (2011). 22114931 10.1186/bcr3039 PMC3315683

[b9] Yuan T. L. & Cantley L. C. PI3K pathway alterations in cancer: variations on a theme. Oncogene 27, 5497–510 (2008). 18794884 10.1038/onc.2008.245 PMC3398461

[b10] Huang C. H. *et al.* The structure of a human p110alpha/p85alpha complex elucidates the effects of oncogenic PI3Kalpha mutations. Science 318, 1744–8 (2007). 18079394 10.1126/science.1150799

[b11] Fruman D. A. & Rommel C. PI3K and cancer: lessons, challenges and opportunities. Nat Rev Drug Discov 13, 140–56 (2014). 24481312 10.1038/nrd4204 PMC3994981

[b12] Urick M. E. *et al.* PIK3R1 (p85alpha) is somatically mutated at high frequency in primary endometrial cancer. Cancer Res 71, 4061–7 (2011). 21478295 10.1158/0008-5472.CAN-11-0549 PMC3117071

[b13] Jaiswal B. S. *et al.* Somatic mutations in p85alpha promote tumorigenesis through class IA PI3K activation. Cancer Cell 16, 463–74 (2009). 19962665 10.1016/j.ccr.2009.10.016 PMC2804903

[b14] Huang Y. *et al.* Multilayered molecular profiling supported the monoclonal origin of metastatic renal cell carcinoma. Int J Cancer 135, 78–87 (2014). 24310851 10.1002/ijc.28654

[b15] Philp A. J. *et al.* The phosphatidylinositol 3'-kinase p85alpha gene is an oncogene in human ovarian and colon tumors. Cancer Res 61, 7426–9 (2001). 11606375

[b16] Taniguchi C. M. *et al.* The phosphoinositide 3-kinase regulatory subunit p85alpha can exert tumor suppressor properties through negative regulation of growth factor signaling. Cancer Res 70, 5305–15 (2010). 20530665 10.1158/0008-5472.CAN-09-3399 PMC3204358

[b17] Cizkova M. *et al.* PIK3R1 underexpression is an independent prognostic marker in breast cancer. BMC Cancer 13, 545 (2013). 24229379 10.1186/1471-2407-13-545 PMC4225603

[b18] Li L., Plummer S. J., Thompson C. L., Tucker T. C. & Casey G. Association between phosphatidylinositol 3-kinase regulatory subunit p85alpha Met326Ile genetic polymorphism and colon cancer risk. Clin Cancer Res 14, 633–7 (2008). 18245521 10.1158/1078-0432.CCR-07-1211

[b19] Qian X. *et al.* N-cadherin/FGFR promotes metastasis through epithelial-to-mesenchymal transition and stem/progenitor cell-like properties. Oncogene 33, 3411–21 (2014). 23975425 10.1038/onc.2013.310 PMC4051865

[b20] Lee J. M., Dedhar S., Kalluri R. & Thompson E. W. The epithelial-mesenchymal transition: new insights in signaling, development, and disease. J Cell Biol 172, 973–81 (2006). 16567498 10.1083/jcb.200601018 PMC2063755

[b21] Kong D., Li Y., Wang Z. & Sarkar F. H. Cancer Stem Cells and Epithelial-to-Mesenchymal Transition (EMT)-Phenotypic Cells: Are They Cousins or Twins? Cancers (Basel) 3, 716–29 (2011). 21643534 10.3390/cancers30100716 PMC3106306

[b22] Jordan C. T. Cancer stem cell biology: from leukemia to solid tumors. Curr Opin Cell Biol 16, 708–12 (2004). 15530785 10.1016/j.ceb.2004.09.002

[b23] Kondo T., Setoguchi T. & Taga T. Persistence of a small subpopulation of cancer stem-like cells in the C6 glioma cell line. Proc Natl Acad Sci U S A 101, 781–6 (2004). 14711994 10.1073/pnas.0307618100 PMC321758

[b24] Baccelli I. & Trumpp A. The evolving concept of cancer and metastasis stem cells. J Cell Biol 198, 281–93 (2012). 22869594 10.1083/jcb.201202014 PMC3413352

[b25] Clevers H. The cancer stem cell: premises, promises and challenges. Nat Med 17, 313–9 (2011). 21386835 10.1038/nm.2304

[b26] Gassenmaier M. *et al.* CXC chemokine receptor 4 is essential for maintenance of renal cell carcinoma-initiating cells and predicts metastasis. Stem Cells 31, 1467–76 (2013). 23630186 10.1002/stem.1407

[b27] Boumahdi S. *et al.* SOX2 controls tumour initiation and cancer stem-cell functions in squamous-cell carcinoma. Nature 511, 246–50 (2014). 24909994 10.1038/nature13305

[b28] Beck B. & Blanpain C. Unravelling cancer stem cell potential. Nat Rev Cancer 13, 727–38 (2013). 24060864 10.1038/nrc3597

[b29] He K. *et al.* Cancer cells acquire a drug resistant, highly tumorigenic, cancer stem-like phenotype through modulation of the PI3K/Akt/beta-catenin/CBP pathway. Int J Cancer 134, 43–54 (2014). 23784558 10.1002/ijc.28341

[b30] Liu C. *et al.* Control of beta-catenin phosphorylation/degradation by a dual-kinase mechanism. Cell 108, 837–47 (2002). 11955436 10.1016/s0092-8674(02)00685-2

[b31] Engelman J. A., Luo J. & Cantley L. C. The evolution of phosphatidylinositol 3-kinases as regulators of growth and metabolism. Nat Rev Genet 7, 606–19 (2006). 16847462 10.1038/nrg1879

[b32] Luo J. & Cantley L. C. The negative regulation of phosphoinositide 3-kinase signaling by p85 and it's implication in cancer. Cell Cycle 4, 1309–12 (2005). 16131837 10.4161/cc.4.10.2062

[b33] Xue G. & Hemmings B. A. PKB/Akt-dependent regulation of cell motility. J Natl Cancer Inst 105, 393–404 (2013). 23355761 10.1093/jnci/djs648

[b34] Bugyi B. & Carlier M. F. Control of actin filament treadmilling in cell motility. Annu Rev Biophys 39, 449–70 (2010). 20192778 10.1146/annurev-biophys-051309-103849

[b35] Zhu Q. S. *et al.* Vimentin is a novel AKT1 target mediating motility and invasion. Oncogene 30, 457–70 (2011). 20856200 10.1038/onc.2010.421 PMC3010301

[b36] Thiery J. P., Acloque H., Huang R. Y. & Nieto M. A. Epithelial-mesenchymal transitions in development and disease. Cell 139, 871–90 (2009). 19945376 10.1016/j.cell.2009.11.007

[b37] Grille S. J. *et al.* The protein kinase Akt induces epithelial mesenchymal transition and promotes enhanced motility and invasiveness of squamous cell carcinoma lines. Cancer Res 63, 2172–8 (2003). 12727836

[b38] Tsukamoto T., Hama S., Kogure K. & Tsuchiya H. Selenate induces epithelial-mesenchymal transition in a colorectal carcinoma cell line by AKT activation. Exp Cell Res 319, 1913–21 (2013). 23769801 10.1016/j.yexcr.2013.05.031

[b39] Yang X., Chrisman H. & Weijer C. J. PDGF signalling controls the migration of mesoderm cells during chick gastrulation by regulating N-cadherin expression. Development 135, 3521–30 (2008). 18832396 10.1242/dev.023416

[b40] Zhou J. *et al.* Activation of the PTEN/mTOR/STAT3 pathway in breast cancer stem-like cells is required for viability and maintenance. Proc Natl Acad Sci U S A 104, 16158–63 (2007). 17911267 10.1073/pnas.0702596104 PMC2042178

[b41] Dubrovska A. *et al.* The role of PTEN/Akt/PI3K signaling in the maintenance and viability of prostate cancer stem-like cell populations. Proc Natl Acad Sci U S A 106, 268–73 (2009). 19116269 10.1073/pnas.0810956106 PMC2629188

[b42] Munoz P., Iliou M. S. & Esteller M. Epigenetic alterations involved in cancer stem cell reprogramming. Mol Oncol 6, 620–36 (2012). 23141800 10.1016/j.molonc.2012.10.006 PMC5528346

[b43] Mani S. A. *et al.* The epithelial-mesenchymal transition generates cells with properties of stem cells. Cell 133, 704–15 (2008). 18485877 10.1016/j.cell.2008.03.027 PMC2728032

[b44] He K., Xu T. & Goldkorn A. Cancer cells cyclically lose and regain drug-resistant highly tumorigenic features characteristic of a cancer stem-like phenotype. Mol Cancer Ther 10, 938–48 (2011). 21518726 10.1158/1535-7163.MCT-10-1120 PMC3112267

[b45] Edge S. B. & Compton C. C. The American Joint Committee on Cancer: the 7th edition of the AJCC cancer staging manual and the future of TNM. Ann Surg Oncol 17, 1471–4 (2010). 20180029 10.1245/s10434-010-0985-4

[b46] Cong L. *et al.* Multiplex genome engineering using CRISPR/Cas systems. Science 339, 819–23 (2013). 23287718 10.1126/science.1231143 PMC3795411

[b47] Qi L. S. *et al.* Repurposing CRISPR as an RNA-guided platform for sequence-specific control of gene expression. Cell 152, 1173–83 (2013). 23452860 10.1016/j.cell.2013.02.022 PMC3664290

