# Supplementary material for: PIK3R1 negatively regulates the epithelial-mesenchymal transition and stem-like phenotype of renal cancer cells through the AKT/GSK3β/CTNNB1 signaling pathway
Source: Sci Rep. 2015 Mar 11;5:8997. doi: 10.1038/srep08997 (PMC4355729; doi:10.1038/srep08997)

PIK3R1 Negatively Regulates the Epithelial-Mesenchymal Transition and Stem-Like Phenotype of Renal Cancer Cells through the AKT/GSK3β/CTNNB1 Signaling Pathway

Youcheng Lin[1*], ZhaoYang[3,4*], Abai Xu[1*], Pei Dong[7], Yi Huang[2], Huan Liu[5], Feida Li[5], Haifeng Wang[9], Qian Xu[10], Yongqiang Wang[2], Da Sun[5], Yong Zou[1], Xiaowen Zou[2], Yu Wang[6], Duo Zhang[5], Hongjie Liu[5], Xun Wu[1], Meng Zhang[2], Yu Fu[2], Zhiming Cai[2], Chunxiao Liu[1§], Song Wu[2,7,8§]

[1]Department of Urology, Zhujiang Hospital of Southern Medical University, Guangzhou 510280, China. [2]National-Regional Key Technology Engineering Laboratory for Clinical Application of Cancer Genomics, Second People's Hospital, the First Affiliated Hospital of Shenzhen University, Shenzhen 518035, China. [3]CAS Key Laboratory of Infection and Immunity, Institute of Biophysics, Chinese Academy of Sciences, Beijing 100101, China. [4]University of Chinese Academy of Sciences, Beijing 100049, China. [5]BGI-Shenzhen, Shenzhen 518083, China. [6]Department of Pathology, Zhujiang Hospital of Southern Medical University, Guangzhou 510280, China. [7]Department of Urology, Sun Yat-Sen University Cancer Center, Guangzhou 510060, China. [8]The Genome Institute, Washington University in St. Louis, 4444 Forest Park Ave, St. Louis, Missouri 63108, USA. [9]Department of Urology, the Second Affiliated Hospital of Kunming Medical College, Kunming 650101, China. [10]Department of Oncology, the First Affiliated Hospital of Soochow University,

Suzhou 215006, China.

Key words: *PIK3R1*, downregulation, migration, stem cell, renal cancer

*These authors contributed equally to this work.

Financial Support: This project was supported by the National Natural Science Foundation of China (Grant No. 81301740) and Innovation Program of Shenzhen (Grant No. JCYJ20130401114715714; No.JCYJ20130401114928183; No. JSGG20130411091246833; No. CXZZ2013051653248144).

§Correspondence and requests for materials should be addressed to**:** Chunxiao Liu, Department of Urology, Zhujiang Hospital of Southern Medical University, Guangzhou 510280, China. Tel: +86-15802037018, Fax: +86-755-83216006, E-mail: liuchx888@163.com; OR to Song Wu, The Genome Institute, Washington University in St. Louis, Louis, Missouri 63108, USA. E-mail: doctor_wusong@126.com

Conflicts of interest: No potential conflicts of interest were disclosed.

Supplementary Table 1. Clinical characteristics of the RCC patients (IHC).

Patient ID	Patient age (years)	Sex	Nephrectomy/ biopsy	Stage (TNM classifcation*)	Sites of metastases
1	68	F	NE	T2aN0M1	bone
2	57	M	BI	T2bN0M1	liver
3	59	F	NE	T1bN0M1	lung
4	56	M	NE	T2aN0M1	brain
5	16	F	NE	T1bN0M1	lung
6	35	F	NE	T1aN1M0	lymph node
7	43	M	BI	T1bN0M1	lung
8	71	F	NE	T1bN1M0	lymph node
9	58	F	NE	T1aN0M1	liver
10	39	F	BI	T3aN0M1	lung
11	56	M	NE	T1bN0M1	lung
12	65	M	BI	T2aN0M1	skin
13	30	M	NE	T1bN1M0	lymph node
14	63	M	BI	T3aN0M1	bone
15	54	M	BI	T2aN0M1	lung and bone
16	24	M	NE	T2aN1M0	lymph node
17	56	F	NE	T1bN0M1	bone
18	48	M	BI	T2aN0M1	lung, bone and liver
19	58	M	NE	T2bN1M0	lymph node
20	62	M	NE	T1aN0M1	bone
21	58	M	BI	T1aN1M1	bone

* The TNM cancer staging system was designed to gauge the extent of cancer in a patient's body. T describes the size of the tumor and whether it has invaded nearby tissue, N describes regional lymph nodes that are involved, and M describes distant metastasis (spread of cancer from one body part to another). NE, nephrectomy. BI, biopsy of metastases. As for two patients (ID 15 and ID 18) who have multiple organ metastases, biopsy of metastases in lung was carried out in the patient (ID 15), whereas biopsy of metastases in bone was carried out in the other patient (ID 18). Metastatic tumor samples of patients who undergo biopsy, were taken from the biopsy metastases, while others were from the resected metastases.

Supplementary Table 2. Clinical characteristics of the RCC patients (RT-PCR).

**Patient ID**	**Patient age (years)**	**Sex**	**Stage (TNM classifcation*)**	**Grade**
1	43	M	T1aN0M0	G3
2	29	F	T1bN0M0	G1
3	49	M	T2aN0M0	NA
4	59	M	T1aN0M0	G3
5	46	M	T1aN0M0	G2
6	64	F	T1aN0M0	G2
7	46	M	T2aN0M0	NA
8	56	F	T1bN0M0	G2
9	44	M	T1aN0M0	G3
10	50	M	T1bN0M0	G3
11	45	M	T2aN0M0	NA
12	65	F	T4NxM1	NA
13	32	F	T1bN0M0	NA
14	62	M	T1aN0M0	G2
15	59	M	T1bN0M0	NA
16	52	M	T1bN0M0	NA
17	39	M	T1aN0M0	NA
18	62	M	T2aN0M1	G3

* The TNM cancer staging system was designed to gauge the extent of cancer in a patient's body. T describes the size of the tumor and whether it has invaded nearby tissue, N describes regional lymph nodes that are involved, and M describes distant metastasis (spread of cancer from one body part to another). NA， not available.

Supplementary Table 3. RT-PCR primer pair sequences for genes.

Gene	Forward primer	Reverse primer
PIK3R1	TGGACGGCGAAGTAAAGCATT	AGTGTGACATTGAGGGAGTCG
ECAD	AAAGGCCCATTTCCTAAAAACCT	TGCGTTCTCTATCCAGAGGCT
NCAD	AGCCAACCTTAACTGAGGAGT	GGCAAGTTGATTGGAGGGATG
VIM	GACGCCATCAACACCGAGTT	CTTTGTCGTTGGTTAGCTGGT
SNAIL	GAAAGGCCTTCAACTGCAAA	TGACATCTGAGTGGGTCTGG
TWIST	GGAGTCCGCAGTCTTACGAG	TCTGGAGGACCTGGTAGAGG
GAPDH	GATGCTGGCGCTGAGTACG	GCTAAGCAGTTGGTGGTGC
CTNNB1	ACAACTGTTTTGAAAATCCA	CGAGTCATTGCATACTGTCC
HES1	TCAACACGACACCGGATAAAC	GCCGCGAGCTATCTTTCTTCA
GLI1	AGAGTAGGGAATCTCATCCATCA	TGATGCAGTTCCTTTATTATCAGG
NANOG	AAGGTCCCGGTCAAGAAACAG	CTTCTGCGTCACACCATTGC
ZEB1	CAGCTTGATACCTGTGAATGGG	TATCTGTGGTCGTGTGGGACT
CD44	CTGCCGCTTTGCAGGTGTA	CATTGTGGGCAAGGTGCTATT
POU5F1	CTTGAATCCCGAATGGAAAGGG	GTGTATATCCCAGGGTGATCCTC

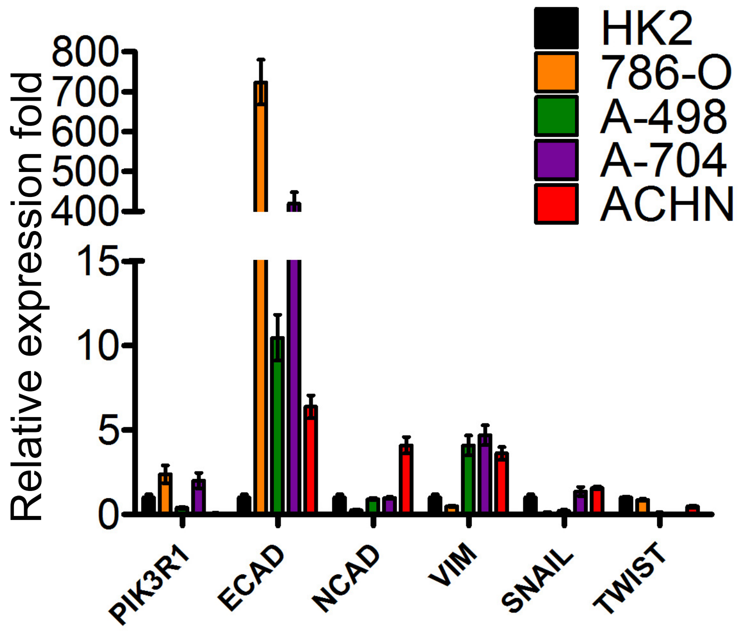

Supplementary Figure 1. RT-PCR analysis of mRNA expression of PIK3R1, ECAD, NCAD, VIM, SNAIL, and TWIST in HK2, 786-O, A-498, A-704, and ACHN cells.

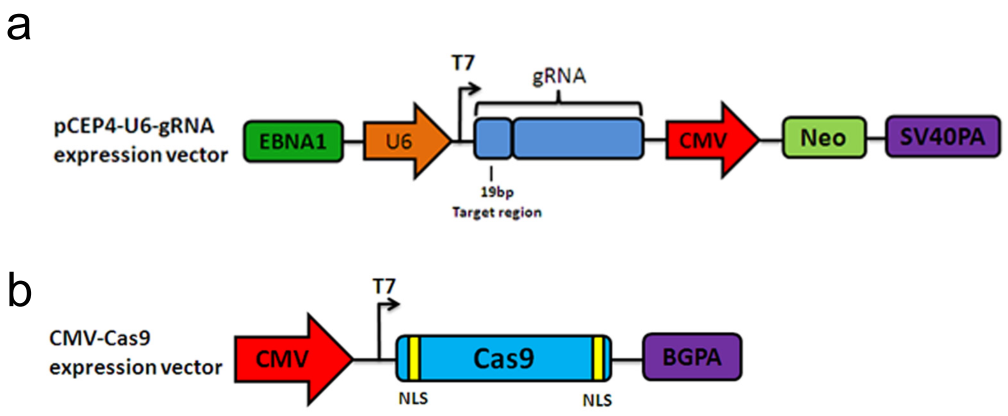

Supplementary Figure 2. Schematic of the pCEP4-U6-gRNA and CMV-Cas9 expression vectors.

a, The U6-gRNA expression cassette including U6 promoter and +83bp sgRNA tails was synthesized and inserted into pCEP4 vector by KpnI and XhoI. The sgRNA was synthesized and inserted into two AarI site between U6 promoter and sgRNA tails to form the pCEP4-U6-gRNA expression vector.

b, The T7 promoter was added upstream of the Cas9 sequence. All the sequences were ligated to the pMD-18T-CMV-MCS-BGPA by NheI/AflII to form CMV-Cas9 expression vector.

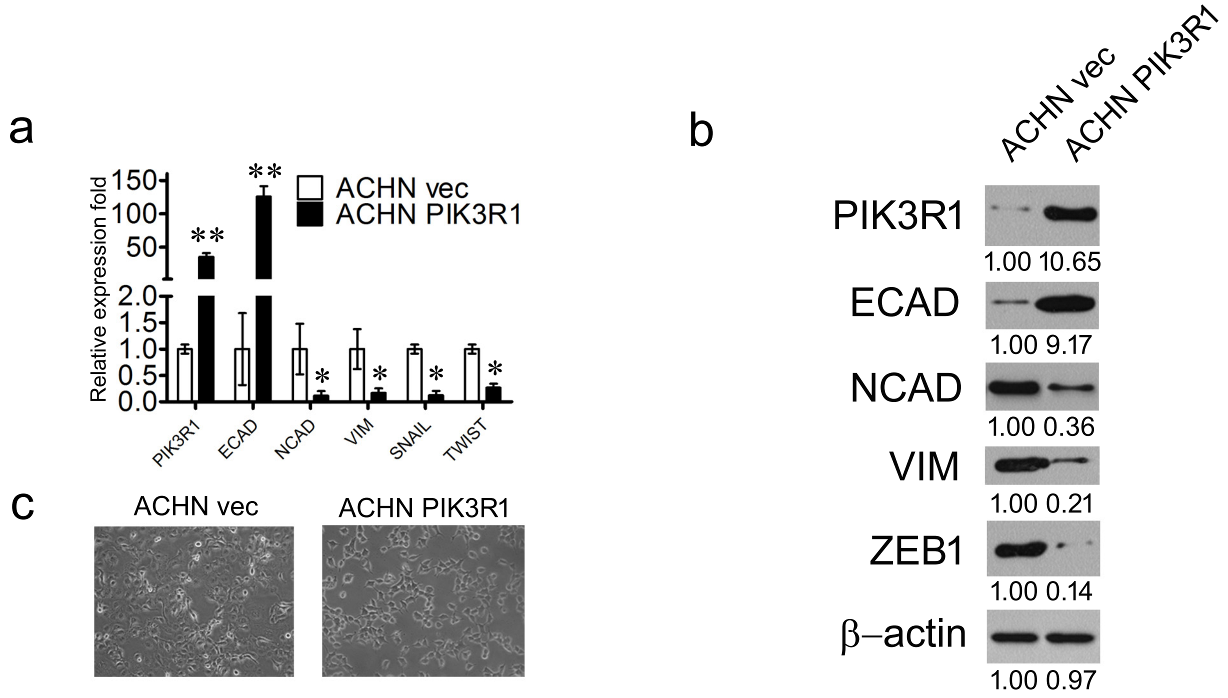

Supplementary Figure 3. Overexpression of PIK3R1 promotes MET in ACHN cells.

a, RT-PCR analysis of PIK3R1, ECAD, NCAD, VIM, SNAIL, and TWIST in ACHN vec and ACHN PIK3R1 cells. b, WB analysis of PIK3R1, ECAD, NCAD, VIM, and ZEB1 protein expression in ACHN vec and ACHN PIK3R1 cells. β-actin was applied as loading control. c, Representative pictures of ACHN vec and ACHN PIK3R1 cells.

Related to Figure 2b

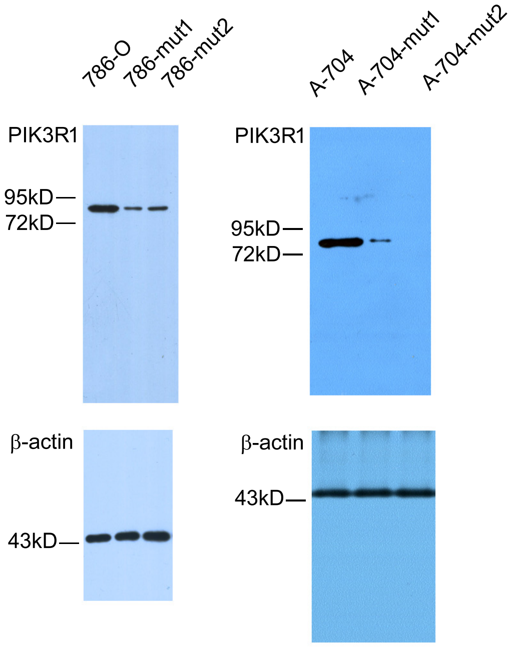

Related to Figure 3d

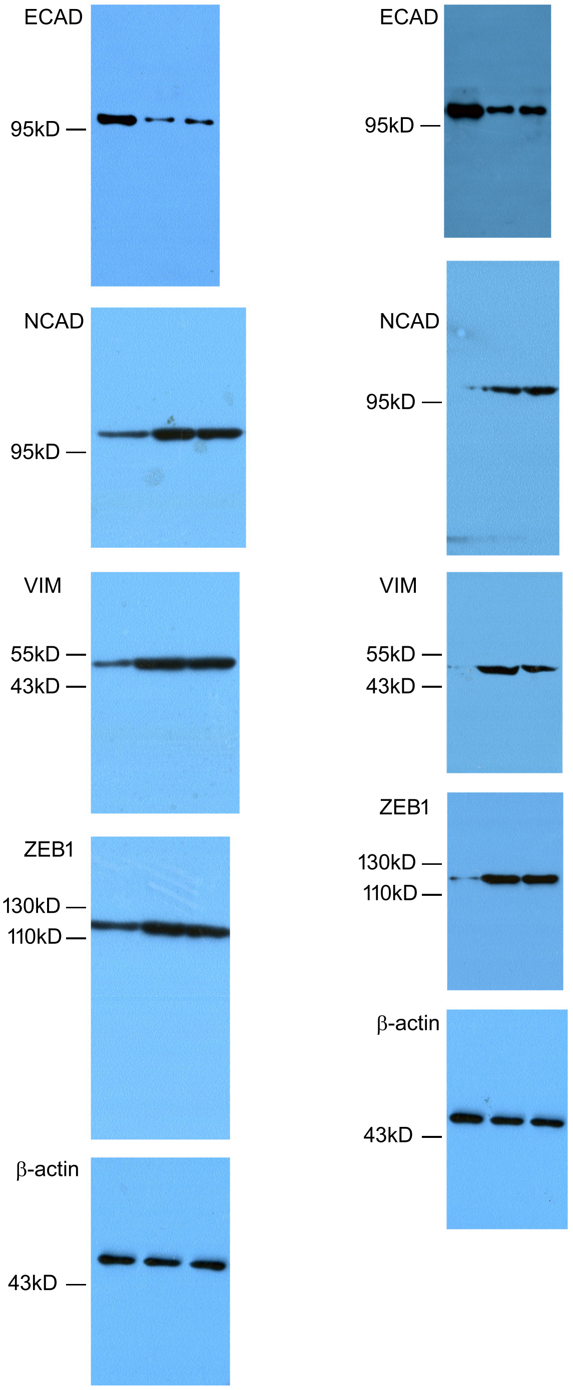

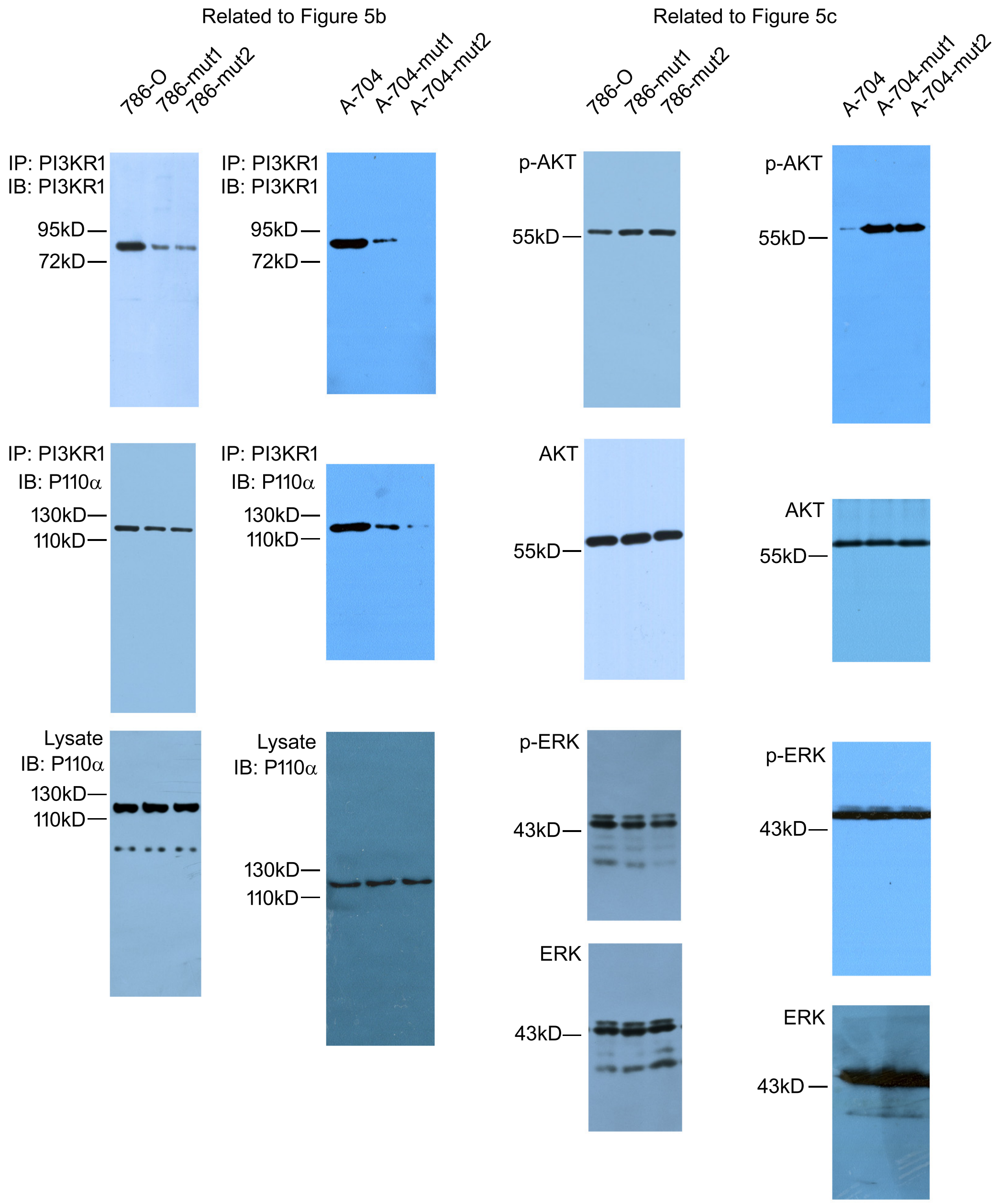

Related to Figure 5b
Related to Figure 5c
786-O
786-mut1
786-mut2
A-704
A-704-mut1
A-704-mut2
IP: PI3KR1
IB: PI3KR1
95kD
72kD
IP: PI3KR1
IB: P110α
130kD
110kD
Lysate
IB: P110α
p-AKT
55kD
AKT
p-ERK
43kD
ERK

Related to Figure 5d

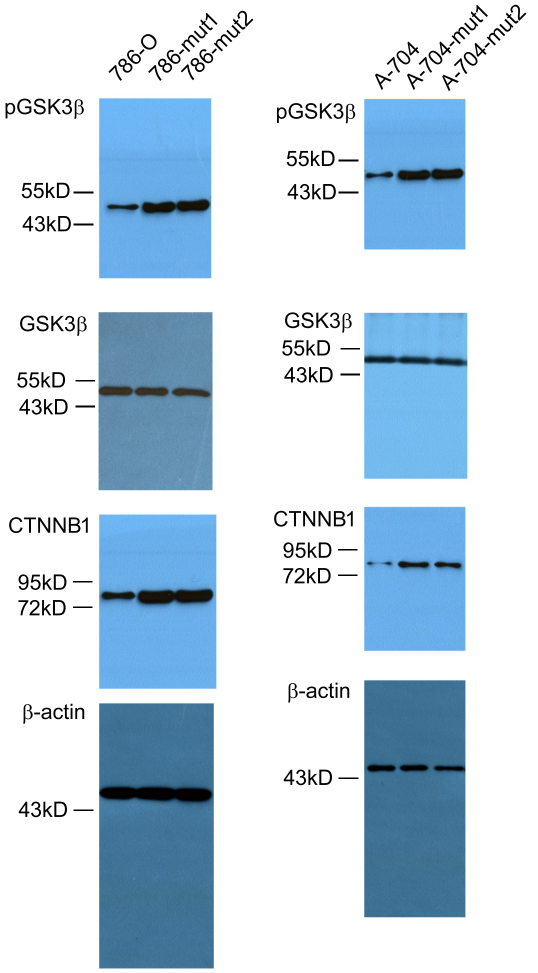

Related to Figure 5e

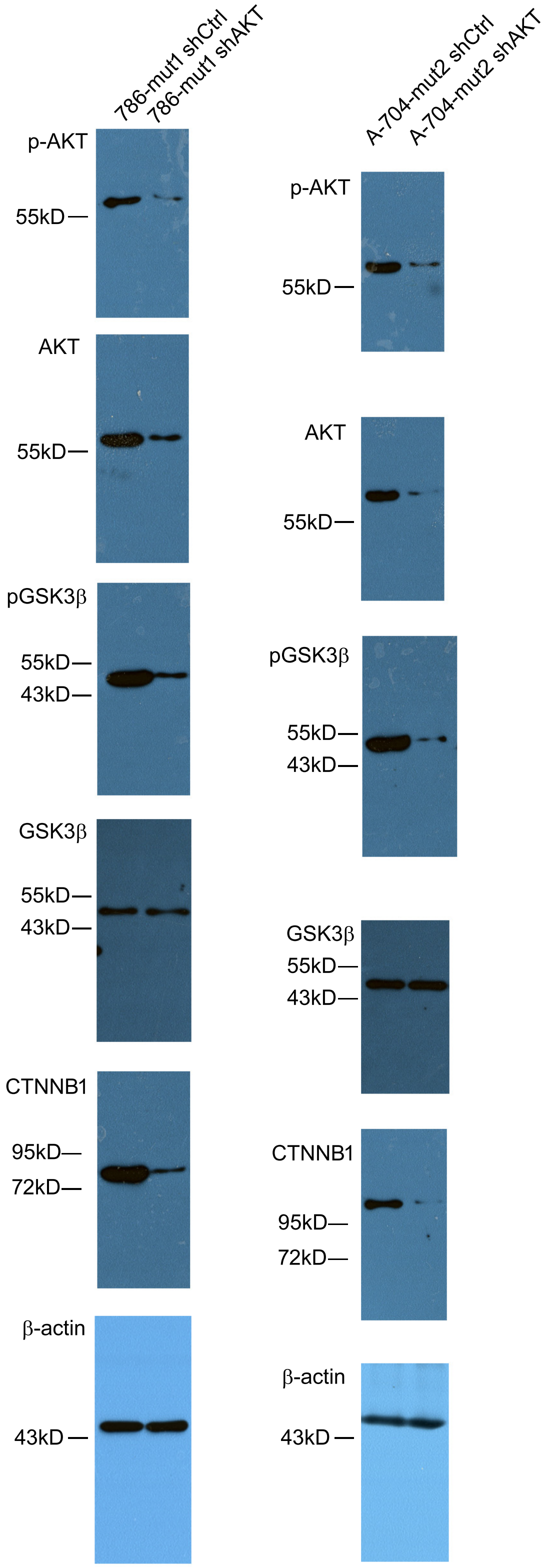

Related to Figure 6a

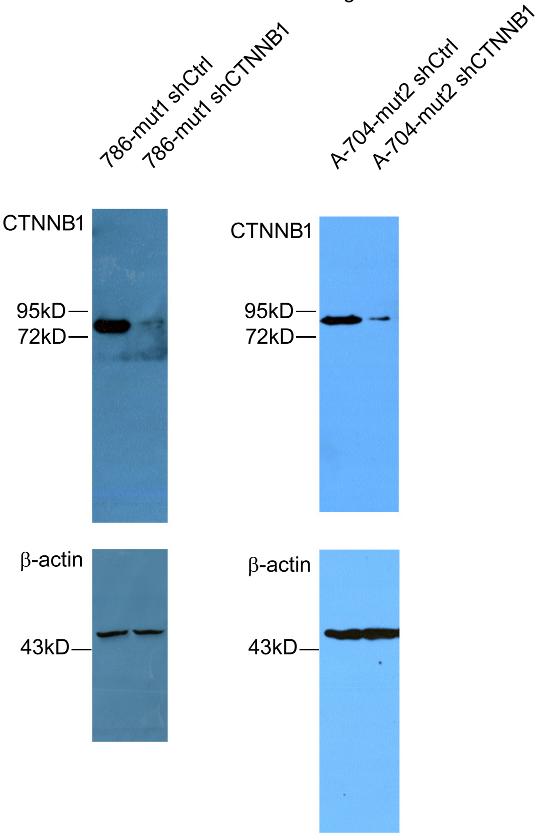

Related to Supplementary Figure 3b

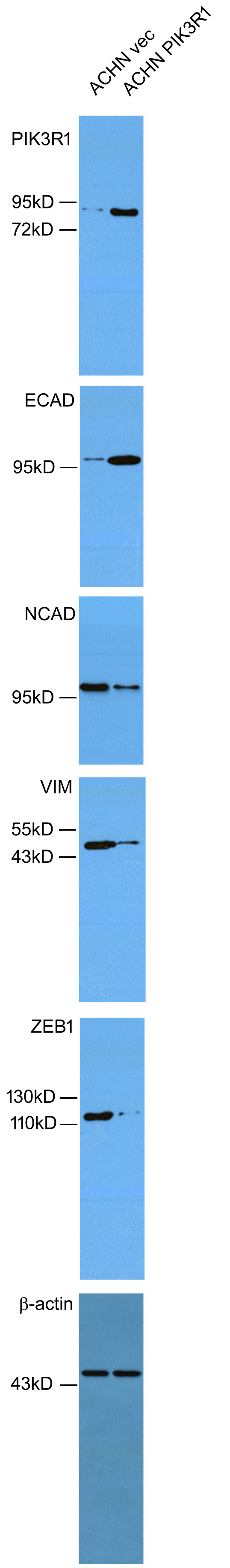

Supplement: Supplementary Information — Supplementary data [file srep08997-s1.pdf]
